# Copper incorporated biomaterial-based technologies for multifunctional wound repair

**DOI:** 10.7150/thno.87193

**Published:** 2024-01-01

**Authors:** Zhenhe Zhang, Hang Xue, Yuan Xiong, Yongtao Geng, Adriana C. Panayi, Samuel Knoedler, Guandong Dai, Mohammad-Ali Shahbazi, Bobin Mi, Gouhui Liu

**Affiliations:** 1 Department of Orthopedics, Union Hospital, Tongji Medical College, Huazhong University of Science and Technology, 1277 Jiefang Avenue, Wuhan 430022, China.; 2 Hubei Province Key Laboratory of Oral and Maxillofacial Development and Regeneration, Wuhan 430022, China.; 3 Division of Plastic Surgery, Brigham and Women's Hospital, Harvard Medical School, Boston, MA 02152, USA.; 4 Department of Hand, Plastic and Reconstructive Surgery, Microsurgery, Burn Center, BG Trauma Center Ludwigshafen, University of Heidelberg, Ludwig-Guttmann-Strasse 13, 67071 Ludwigshafen/Rhine, Germany.; 5 Institute of Regenerative Biology and Medicine, Helmholtz Zentrum München, Max-Lebsche-Platz 31, 81377 Munich, Germany.; 6 Department of Orthopaedics, Pingshan District People's Hospital of Shenzhen, Pingshan General Hospital of Southern Medical University, Shenzhen 518118, China.; 7 Department of Biomedical Engineering, University Medical Center Groningen, University of Groningen, Antonius Deusinglaan 1, 9713 Groningen AV, The Netherlands.; 8 W.J. Kolff Institute for Biomedical Engineering and Materials Science, University of Groningen, University Medical Center Groningen, Antonius Deusinglaan 1, 9713 Groningen AV, The Netherlands.

**Keywords:** Copper, Antibacterial, Nanozyme, Photothermal therapy, Photodynamic therapy

## Abstract

The treatment of wounds is a worldwide challenge, and wound infection can affect the effectiveness of wound treatment and further increase the disease burden. Copper is an essential trace element that has been shown to have broad-spectrum antibacterial effects and to be involved in the inflammation, proliferation, and remodeling stages of wound healing. Compared to treatments such as bioactive factors and skin grafts, copper has the advantage of being low-cost and easily available, and has received a lot of attention in wound healing. Recently, biomaterials made by incorporating copper into bioactive glasses, polymeric scaffolds and hydrogels have been used to promote wound healing by the release of copper ions. In addition, copper-incorporated biomaterials with catalytic, photothermal, and photosensitive properties can also accelerate wound healing through antibacterial and wound microenvironment regulation. This review summarizes the antibacterial mechanisms of copper- incorporated biomaterials and their roles in wound healing, and discusses the current challenges. A comprehensive understanding of the role of copper in wounds will help to facilitate new preclinical and clinical studies, thus leading to the development of novel therapeutic tools.

## Introduction

The skin is an organ in direct contact with the external environment and its integrity is often compromised by trauma, burns and surgery 1. Wound healing requires the concerted cooperation of multiple cells that undergo hemostasis, inflammation, proliferation, and remodeling phases to restore skin integrity 2. However, diabetes and bacterial infections tend to alter the wound microenvironment, causing local hypoxia, accumulation of reactive oxygen species (ROS), and reduced expression of growth factors, leading to dysregulated inflammation, impaired angiogenesis, and reduced collagen production, resulting in non-healing wounds and, in severe cases, amputation or death 3. It has been reported that the prevalence of chronic wounds is as high as 2%, requiring tens of billions of dollars to be spent on wound management each year 4. Although autografts, allografts, xenografts, and bioengineered skin are potential treatments for skin defects, they have disadvantages such as disease transmission, biological rejection, and preparation difficulties 5. With an aging population and an increasing number of people with diabetes, the incidence of chronic wounds will further increase, making it urgent to find more effective wound management methods 3.

Various metals, including copper, silver, gold, and zinc, have long been utilized in the treatment of infections. Among these antibacterial metals, copper stands out for its ability to achieve a balance between antibacterial efficacy and biocompatibility 6. It not only demonstrates significantly greater antibacterial capacity than metals like zinc and gold but also boasts a higher cellular tolerance concentration compared to silver and zinc 6, 7. In addition, compared to gold and silver, copper is more suitable for clinical translation due to its abundant natural content, ready availability and cost-effectiveness 8. Especially in recent years, copper has been found to play a role in processes such as angiogenesis, collagen formation and matrix remodeling, further making it an ideal metal for treating wounds 9.

Despite the multifunctional properties of copper driving its application in wound care, its use in wound therapy has long been restricted to copper ions or copper oxides 10, 11. This approach primarily relies on increasing the local concentration of copper ions to exert antibacterial and cellular function modulation effects. With the progress of nanomedicine, many copper-containing nanomaterials with high porosity, high specific area, and mimetic enzyme-like activity have been constructed for drug delivery, ROS scavenging and enhanced antibacterial efficacy, making them highly promising for wound treatment 12, 13. In the past five years, the photothermal and photosensitive properties of copper-containing biomaterials have received widespread attention, leading to the proposal of wound treatment strategies based on bacterial clearance such as targeted photothermal antibacterial and low-temperature photothermal therapy, bringing wound treatment into the era of precision 14, 15.

Although copper-containing nanomaterials are multifunctional, the treatment of wounds requires specific media as wound dressings in order to exert wound protection and create a suitable environment for wound repair. Therefore, utilizing bioactive glass, polymer and hydrogel scaffolds for copper loading is considered an effective solution 16-18. They can act as carriers for multifunctional copper nanomaterials, stabilizing the nanoparticles and sustaining the release of Cu^2+^, reducing the frequency of administration. In addition, these copper-containing composites are able to exhibit their own unique advantages in wound healing. For example, bioactive glass can release silicate ions and calcium ions, which together with copper ions modulate the function of cellular networks during wound healing 19. Polymers can be prepared as nanofiber scaffolds to mimic the extracellular matrix to support cellular function 20. And hydrogels can absorb exudate, moisturize, reduce wound temperature, relieve pain, and stop bleeding 21. Overall, the introduction of these matrices has greatly enriched the functionality of copper-containing biomaterials, making them more compatible with the needs of wound therapy.

So far, various wound healing strategies based on copper-containing biomaterials have been developed, but a comprehensive summary of their application in wounds is still lacking. This review summarizes the antibacterial mechanisms of copper-containing biomaterials in wounds in the context of the microenvironment of wounds. Then, this review provides a comprehensive overview of the role played by copper in the different stages of wound healing. Subsequently, as mentioned above, since nanoparticles, bioactive glass, polymer scaffolds, and hydrogel scaffolds show different characteristics in wound treatment, this review summarizes the application of copper in wounds using this classification. In addition, since copper-containing photo-responsive materials have been extensively studied in recent years and have spawned a variety of therapeutic strategies, they are summarized in a separate section to allow for a better understanding of progress. Finally, this review discusses the biocompatibility issues and the construction directions of copper-containing biomaterials to provide guidance for subsequent research and clinical translation of copper-containing biomaterials.

## Antibacterial effect of copper-incorporated biomaterials

**Figure 1:**
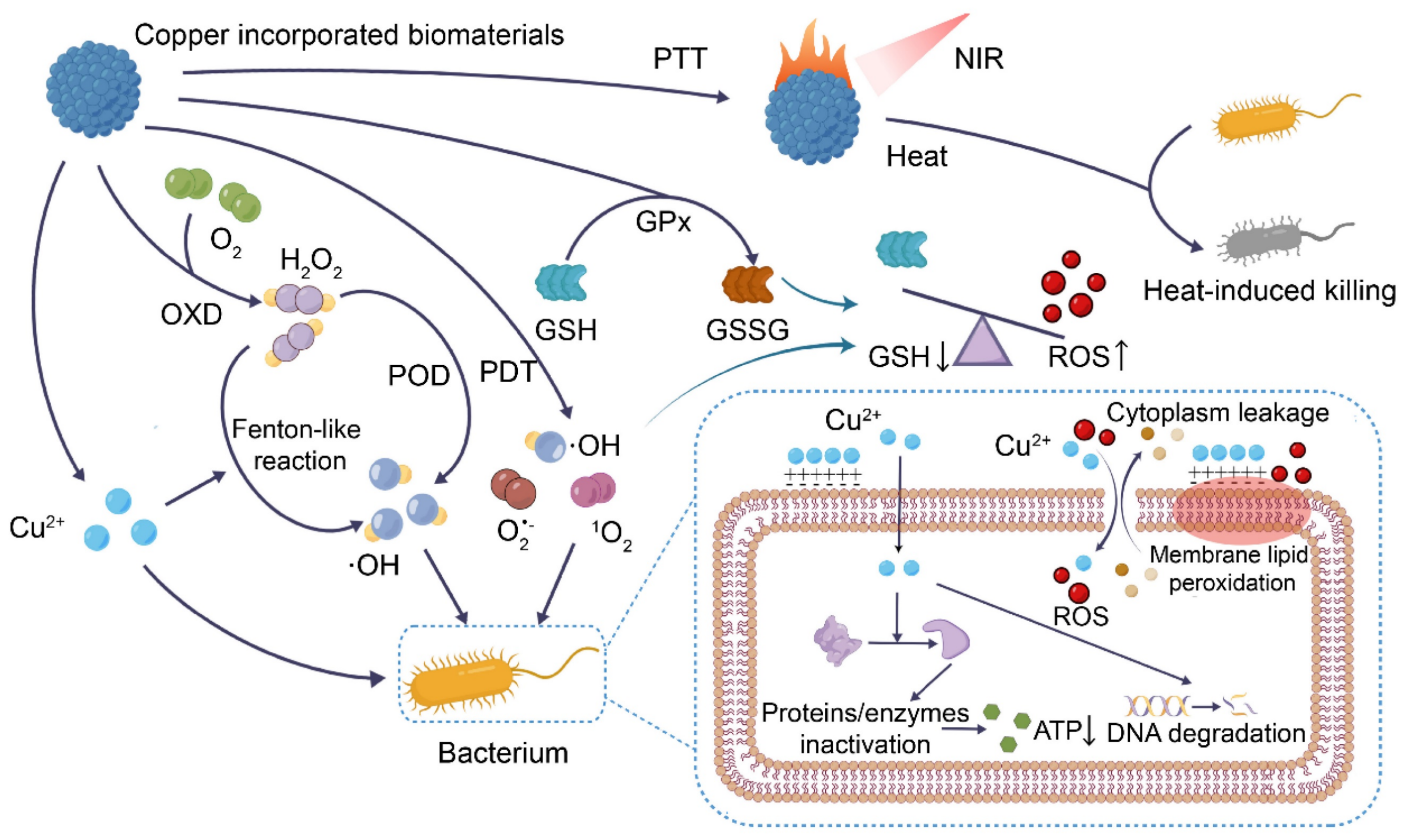
Antibacterial mechanisms of copper-incorporated biomaterials. The biomaterials release Cu^2+^, which induces bacterial membrane lipid peroxidation and increases bacterial cell membrane permeability, leading to the efflux of cell contents. At the same time, Cu^2+^ and ROS enter the cell to inactivate proteins and enzymes, impair energy metabolism, and degrade DNA. Copper-incorporated biomaterials can also deplete bacterial glutathione (GSH), and generate hydroxyl radicals (·OH), superoxide anion radicals (O_2_^•-^) and singlet oxygen (^1^O_2_) via Fenton-like reaction, enzyme-like activity, and photodynamic therapy (PDT), leading to bacterial oxidative stress imbalance. Copper-incorporated photothermal materials also kill bacteria by raising the local temperature through photothermal conversion. (POD, peroxidase-like activity; OXD, oxidase-like activity; GPx, GSH peroxidase-like activity; PTT, photothermal therapy).

When the skin is damaged, bacteria tend to invade and proliferate, triggering a constant and high level of inflammation that prevents the wound from healing 2. Copper has been used as an antibacterial agent since ancient times, not only to kill microorganisms including drug-resistant bacteria and fungi but also to inhibit biofilm formation 22. Since human skin cells are less susceptible to copper than microorganisms, proper concentrations of copper can limit microbial growth without harming healthy cells 23. In vivo studies have found a low risk of adverse reactions following direct skin contact with copper, additionally inferring that copper is safe for antibacterial use in wounds 24. Here, the antibacterial mechanisms of copper-containing biomaterials are summarized (Figure 1).

### Direct destruction of molecular and cellular structures

The cell membrane prevents extracellular substances from freely entering the cell and ensures the stability of the intracellular environment, which is the basis for cell survival 25. Cu^2+^ released from copper-containing biomaterials can electrostatically bind to negatively charged bacterial cell membranes 23, 26. The positive charge of Cu^2+^ changes the cell membrane potential and augments its permeability, thus, leading to structural damage of cell membranes and leakage of cellular contents 23.

Due to the increased permeability of the bacterial cell membrane and the small hydration radius of Cu^2+^ (87 × 10^-3^ nm), a large amount of Cu^2+^ can enter the cell 27. In addition, the intracellular release of Cu^2+^ from copper-containing nanoparticles (NPs) that penetrate cell membranes or are swallowed by cells is also important for the accumulation of high levels of Cu^2+^ in bacterial cells 26, 28, 29. High levels of intracellular Cu^2+^ can affect the biological activities of proteins by binding protein functional groups or by disrupting protein structure 30-32. Cu^2+^ also has a high affinity for sulfur- and phosphorus-rich DNA, causing oxidative damage to DNA, destroying the supercoiled structure of DNA, and causing DNA breakage 26, 29, 33. In addition, by replacing other metal ions in the ligand, Cu^2+^ also interferes with ATP synthesis-related enzyme activities, impeding ATP synthesis and ultimately inducing cell death 34.

It is worth noting that a new form of cell death dependent on copper has recently been discovered, known as cuproptosis 35. Specifically, excess intracellular Cu^2+^ binds to lipoylated components in the tricarboxylic acid (TCA) cycle, leading to these protein aggregation and iron-sulfur clusters reduction, which induces proteotoxic stress and ultimately cell death. A recent study found that promoting bacterial metabolism can enhance the accumulation of copper-containing nanomaterials inside bacteria and cause copper overload, thereby inhibiting the bacterial TCA cycle, and ultimately leading to bacterial cuproptosis-like death 36. Undoubtedly, studies on bacterial cuproptosis-like death will help to further understand the antibacterial mechanism of copper. In addition, it will promote the construction of copper-based biomaterials based on the "cuproptosis-like death" antibacterial strategy, and promote their antibacterial application in wounds.

### Fenton-like reaction

The toxicity of copper is also related to the ROS generated by copper alternating between dissimilar valence states 37. Cu^2+^ released from copper-containing biomaterials can be converted to Cu^+^ by either endogenous or exogenous glutathione (GSH) 38. Cu^+^ has shown an ability to undergo Fenton-like reactions with H_2_O_2_ to generate hydroxyl radicals (·OH) and Cu^2+^ 39. In infected wounds, higher H_2_O_2_ levels contribute to Fenton-like reactions in copper-containing materials, generating more ·OH 26, 40. ROS level that exceeds the scavenging capacity of bacteria induces bacterial membrane damage, DNA degradation, and membrane lipid peroxidation, leading to bacterial death 12, 41. Of note, copper-based Fenton-like reaction systems catalyze the generation of ·OH at a faster rate and operate over a broader pH range compared to iron-based Fenton reaction systems 42. Therefore, they hold a distinct advantage in bacterial eradication.

### ROS production and GSH consumption by nanozymes

Nanozymes are nanomaterials with enzyme-mimetic activity 43. Nanoparticles made of some metals and metal oxides have been shown to have peroxidase (POD), oxidase (OXD), catalase (CAT), and superoxide dismutase (SOD)-like activities for the regulation of ROS levels 44. Currently, copper-containing nanozymes can attack bacteria by mimicking OXD, which converts O_2_ to H_2_O_2_, and POD, which converts H_2_O_2_ to more toxic ·OH 45. The material releases copper ions, which induce bacterial membrane lipid peroxidation and increase bacterial cell membrane permeability, leading to the efflux of cell contents, while at the same time, copper ions and ROS enter the cell to inactivate proteins and enzymes, impair energy metabolism, and degrade DNA.

GSH, a tripeptide with antioxidant activity, accumulates substantially at the site of infection due to anaerobic glycolysis, thereby reducing the antibacterial effect of ROS 46, 47. Copper-containing nanozymes with glutathione peroxidase (GPx)-like activity are able to deplete wound GSH, ensuring the antibacterial effect of copper-containing biomaterials 48, 49.

### Photothermal and photodynamic therapy

Photothermal agents absorb light at specific wavelengths and convert light energy into heat 50. This results in the thermal ablation of cells and is often used in photothermal therapy (PTT) for malignant tumors 51. Photodynamic therapy (PDT) refers to the production of superoxide anion radicals (O_2_^•-^) by type I photodynamic reaction or singlet oxygen (^1^O_2_) by type II photodynamic reaction of photosensitizers under specific wavelength laser irradiation 52, 53. In recent years, copper sulfide (CuS) has been used as a photothermal and photosensitizer for PTT and PDT at wounds due to its low toxicity, low cost, and stable photothermal and photodynamic properties 54. After irradiation of the wound with a near-infrared (NIR) laser, CuS increases the local temperature of the wound, and generates O_2_^•-^ and ^1^O_2_ with strong oxidizing properties to destroy the biofilm structure, and kill bacteria 53, 55. Recently, a "hot ions effect" has also been identified, whereby the local increase in temperature caused by laser irradiation of copper-containing photothermal materials leads to a significant increase in the antibacterial capacity of Cu^2+^ 56. The mechanism may be related to the increase in bacterial membrane permeability at elevated temperatures 56, 57.

## Copper and wound healing

### Mechanism of wound healing

Immediately after injury, damaged blood vessels constrict and platelets are activated and aggregated to form a blood clot consisting of platelets and fibrin to stop the wound from bleeding 58. Inflammatory cells such as neutrophils and macrophages are recruited to the wound by chemokines to remove necrotic tissue and bacteria through phagocytosis, the release of inflammatory cytokines, and proteolytic enzymes 59. In the subsequent proliferation phase, fibroblasts migrate to the wound, produce collagen, and some differentiate into myofibroblasts, which drive wound contraction 60. Endothelial cells are stimulated by signals such as vascular endothelial growth factor (VEGF) and fibroblast growth factor (FGF)-2 to proliferate and form blood vessels in the wound to provide oxygen and nutrients to the wound 61. The wound remodeling phase lasts for several years after the injury and is characterized by a progressive increase in type III collagen in the tissue, accompanied by a progressive decrease in type I collagen, during which matrix metalloproteinases (MMP) play a major regulatory role 62. The remodeling of the wound makes the collagen fibers more closely arranged, the tissue strength increases, and the extracellular matrix structure gradually approaches the structure of normal skin tissue 63.

Under the influence of factors such as high glucose and infection, the wound healing cascade is disrupted, resulting in chronic wounds. Specifically, repeated tissue injury, microbes, and high levels of glucose overactivate inflammatory cells, leading to high interleukin (IL)-1β and tumor necrosis factor (TNF)-α expression and ROS accumulation 64. Prolonged high levels of inflammation and oxidative stress at the wound site further lead to decreased proliferation and functionality of fibroblasts, keratinocytes, and endothelial cells, imbalance of MMP and tissue inhibitor of metalloproteinase (TIMP), degradation of growth factors and their receptors, resulting in impaired vascular formation, inadequate oxygen supply, disruption of extracellular matrix synthesis and remodeling, and compromised re-epithelialization 65.

In terms of treatment, although copper-containing biomaterials have achieved good results in the treatment of both acute and chronic wounds, their therapeutic mechanisms are different. It has long been recognized that acute wounds can heal without special treatment, but study has shown that the use of copper in acute wound accelerates wound healing 9. This is because copper ions can promote the proliferation and migration of endothelial cells, fibroblasts and keratinocytes, accelerate angiogenesis, granulation tissue formation, collagen deposition and re-epithelialization in acute wounds, and ultimately make wounds heal in a shorter period of time. In chronic wounds, some copper-containing biomaterials are first considered to remove the cause. For example, copper-containing materials used in the treatment of infected wounds often give priority to the antibacterial properties of the material 66. In addition, copper-containing materials would also focus on reversing unfavorable pathological features in chronic wounds, such as suppressing excessive inflammation by modulating immunity or scavenging excessive ROS by mimicking enzyme activity 67, 68. The released copper ions are then used to promote angiogenesis, granulation tissue formation, collagen deposition and re-epithelialization, thus aiding in chronic wound healing.

**Figure 2:**
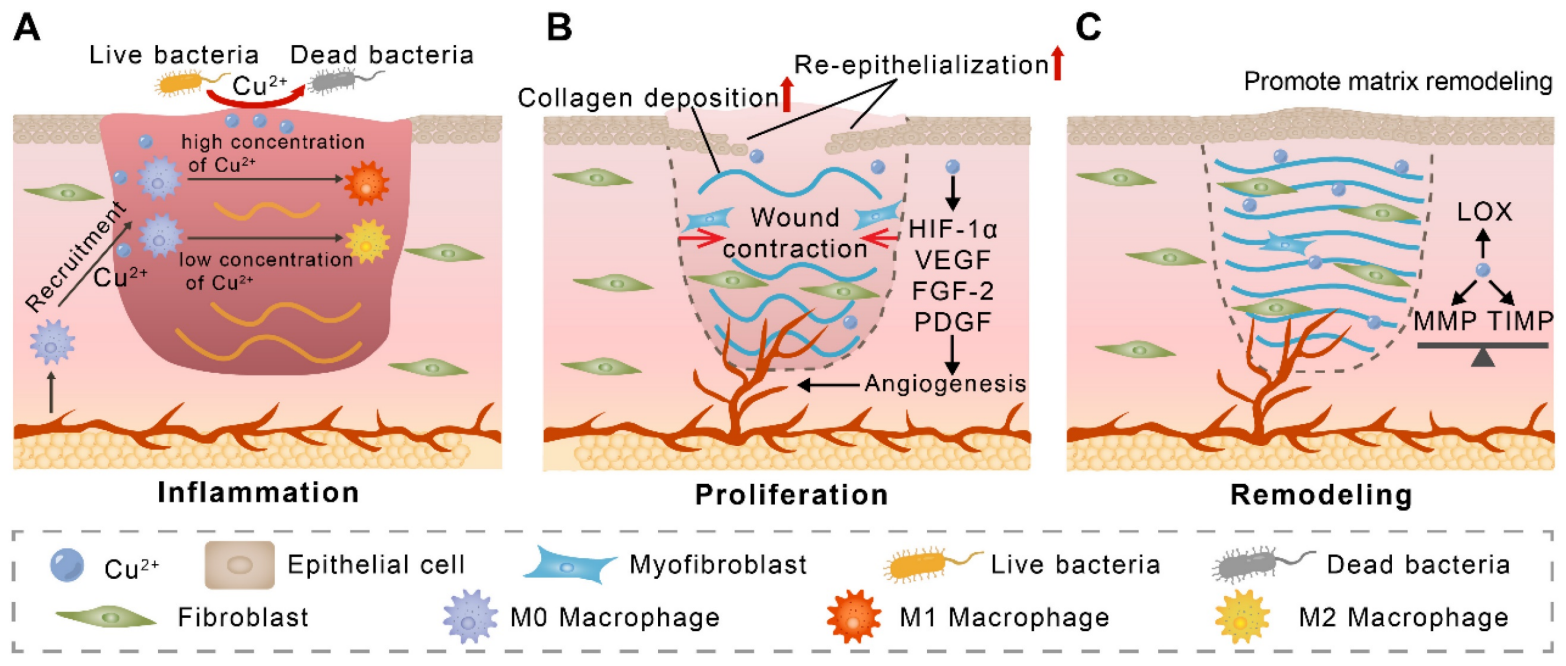
The proposed mechanism of copper-mediated wound healing acceleration. (A) M0 macrophages are recruited to the wound by Cu^2+^ and subsequently polarized to M1 macrophages in response to high concentrations of Cu^2+^ or to M2 macrophages in response to low concentrations of Cu^2+^. (B) During the proliferation phase, copper promotes angiogenic factor expression and wound angiogenesis. Copper also accelerates wound contraction and re-epithelialization. (C) Copper maintains the balance of MMP and TIMP and acts as a cofactor for lysyl oxidase (LOX), which facilitates wound matrix remodeling.

Given the versatility of copper in wound healing, we summarize the regulatory role of copper in wound healing in this section (Figure 2). The application of copper nanomaterials and copper-containing composite biomaterials in wounds will be discussed in subsequent sections.

### Copper and inflammation

Inflammatory modulation is critical for tissue repair. Recently, the regulation of macrophages by copper ions has received much attention. Copper ions have been reported to promote the migration of Raw 264.7 macrophages 69. Some in vivo studies have shown that placing copper-containing implants leads to more CD68^+^ macrophage infiltration in the surrounding soft tissue 70, 71. These studies suggest that copper contributes to the recruitment of M0 macrophages to tissue injury. Currently, there are many studies pointing to the ability of copper ions to modulate macrophage polarization. However, the results of these studies are contradictory. For example, Xiao et al. found that wound tissue exhibited more M1 macrophages on the third day after treatment of wounds with copper-containing dressings 72. In another study, Xu et al. found that copper decreased macrophage pro-inflammatory gene expression and increased anti-inflammatory gene expression 73. While Jian et al. found that copper-containing hydrogel-treated macrophages exhibited more M1 polarization on day 3 and more M2 polarization on day 7 74. This inconsistency may be due to the fact that the regulation of macrophage polarization by copper ions is concentration-dependent, as a recent in vitro study found that treatment of the THP-1 monocyte cell line with Cu^2+^ at concentrations lower than 10 μM for 48 h promoted the expression of M2 macrophage-associated markers, whereas Cu^2+^ at concentrations higher than 100 μM increased M1 macrophage-associated marker expression 75. Notably, polymorphonuclear granulocytes, MHC-II^+^ cells, and T lymphocytes may also be regulated by copper ions, although more studies are needed to confirm this 71, 76.

### Copper and angiogenesis

Neovascularization transports oxygen and nutrients, removes metabolic waste, and promotes wound healing 77. In vivo studies have shown that copper treatment promotes angiogenesis, which is related to the ability of copper to promote endothelial cell proliferation and migration 9, 11, 78, 79. In one study, copper deficiency induced by tetrathiomolybdate significantly reduced the expression of pro-angiogenic factors and inhibited angiogenesis, further confirming the role of copper in promoting angiogenesis 80. Copper promotes angiogenesis through several pathways, with copper-dependent VEGF expression being one of the important mechanisms. This can be achieved by copper stimulation of insulin-like growth factor-1 expression and upregulation of hypoxia-inducible factor (HIF)-1α expression 81, 82. Copper-regulated VEGF is involved in several aspects of angiogenesis, including induction of migration and dynamic transformation of endothelial cells (tip cells and stalk cells), increase in vascular permeability and regulation of lumen diameter 83. Moreover, it has been shown that placental growth factor levels can be upregulated by copper through activation of VEGF receptor-1 (VEGFR1) and synergistic enhancement of VEGF, thereby promoting angiogenesis 81, 84. In addition to VEGF, copper modulates the expression and activity of several angiogenic factors, such as angiogenin (ANG), FGF, and platelet-derived growth factor, which collectively regulate angiogenic processes 11, 16. For example, copper significantly upregulates the expression of ANG, which acts as an angiogenic agent by promoting basement membrane degradation, stimulating signaling, and exerting ribonucleolytic activity 85. Copper acts as a transport cofactor for FGF-1 and promotes its release, thereby stimulating endothelial cell migration. Copper also plays a key role in angiogenesis by regulating the expression of endothelial nitric oxide synthase, which promotes the proliferation of endothelial cells, fibronectin, which promotes vessel elongation, and elastin, which forms and maintains the lumen 83.

### Copper and wound contraction and re-epithelialization

Early contraction of the wound helps to reduce the area of exposed skin, thus reducing the risk of infection 86. Copper treatment has been shown to accelerate centripetal contraction of the wound edges, thereby reducing wound healing time 10, 87. Myofibroblasts are the main driver of wound contraction 88. Although copper promotes the number of fibroblasts at the wound site, whether its promotion of wound contraction is related to the promotion of fibroblast to myofibroblast differentiation remains to be investigated 89.

The epidermis consists of multiple layers of keratinocytes 90. In the process of wound healing, keratinocytes migrate to the surface of the wound and form a tight epidermal barrier through intercellular connections (re-epithelialization) 91. Copper has been shown to upregulate the expression of integrin α2, integrin α6, and integrin β1, promoting keratinocytes migration and epidermal-dermal junction 92, 93. In animal wound models, more epidermal cells and more intact epidermal structures are observed in copper-treated wounds, representing that copper promotes re-epithelialization of wounds 16, 94.

### Copper and extracellular matrix deposition and remodeling

Wound remodeling involves the deposition and remodeling of the extracellular matrix, with fibroblasts dominating this process 95. Copper has been shown to directly enhance fibroblast migration in vitro 93, 96. In vivo studies have shown more fibroblasts in copper-treated wounds, which may be related to the fact that copper stimulates more MMP to degrade the matrix and further enhance the migration of fibroblasts 89, 97, 98. In response to copper stimulation, fibroblasts increase the expression of extracellular matrix components such as type I collagen, dermatan sulfate, chondroitin sulfate, and decorin 99, 100. Copper further promotes the expression of the TGF-β1, which acts on fibroblasts and increases the expression of collagen and fibronectin 101.

Degradation of the extracellular matrix by MMP is an important process in matrix remodeling 102. Copper has been shown to increase the activity or expression levels of MMP1, MMP2, and MMP9 in fibroblasts 96, 103. In contrast, TIMP has been shown to regulate the function of MMP and prevent excessive degradation of the matrix by MMP 104. Copper was found to promote MMP expression while similarly increasing the secretion of TIMP-1 and TIMP-2 97. This plays an important role in the balance between TIMP and MMP 96, 97, 103. In addition, copper acts as a cofactor for LOX and is involved in the regulation of collagen and elastin cross-linking 105. Overall, in vivo studies have shown that copper-treated wounds have more collagen deposition, more ordered collagen fiber arrangement, and a similar structure to normal skin, further demonstrating that copper regulates extracellular matrix remodeling and aids in wound healing 16, 94.

## Copper-incorporated nano-biomaterials

**Figure 3:**
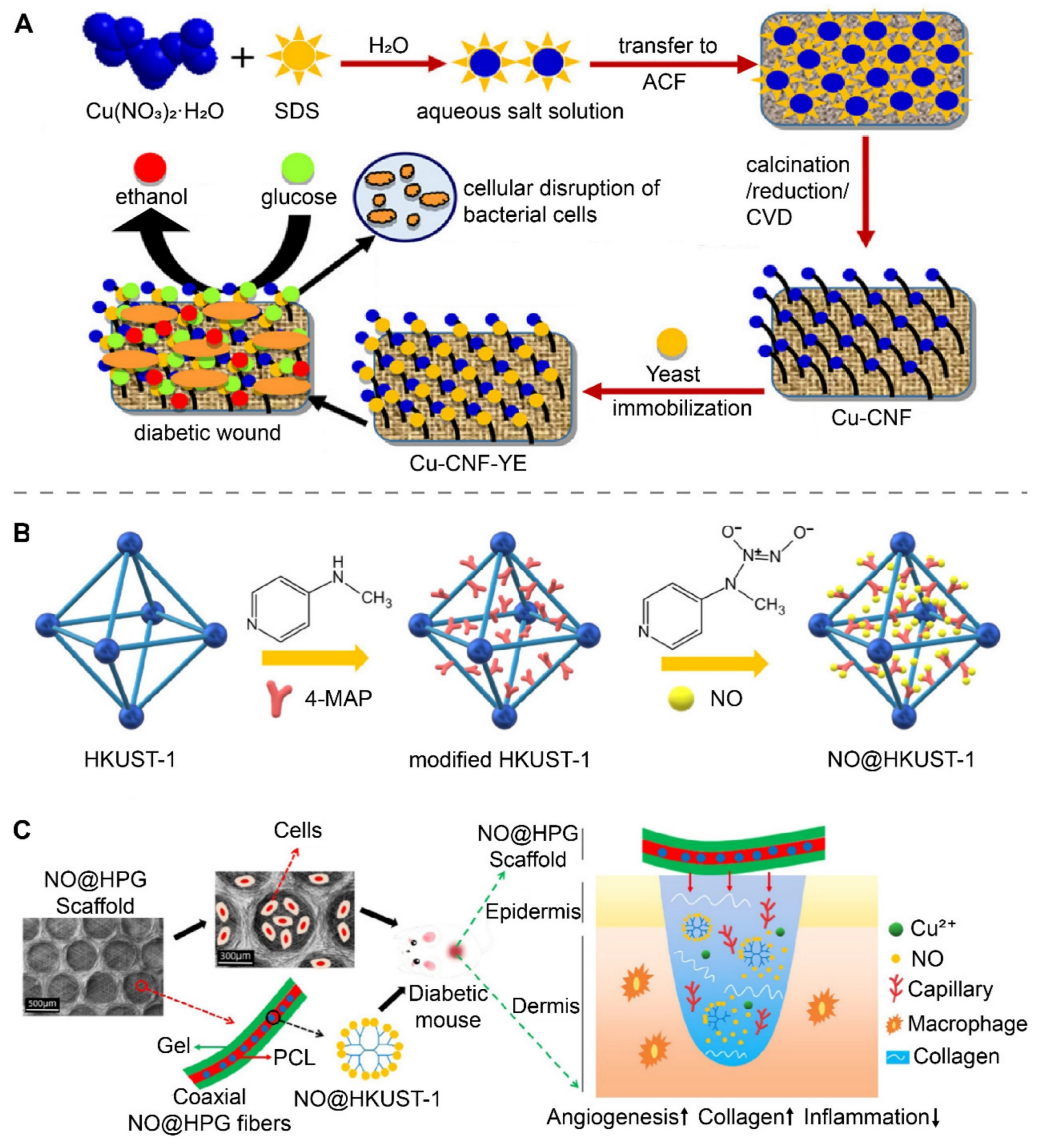
Copper-containing nanomaterials doped with bioactive substances for wound treatment. (A) Synthesis and mechanism schematic illustration of the Cu-CNF-YE for diabetic wound healing. Reproduced with permission from 106. Copyright 2018, American Chemical Society. (B) HKUST-1 modified with secondary amine groups for loading NO. (C) Schematic illustration of NO@HPG scaffolds for accelerating diabetic wound healing. Reproduced with permission from 13. Copyright 2020, American Chemical Society.

Cu and CuO nanoparticles, and materials based on these nanoparticles have been shown to exert antibacterial and accelerated wound-healing effects in vivo 9, 89. The incorporation of other bioactive substances into copper-containing nanomaterials can provide additional strategies to treat wounds. Recently, Bhadauriya et al. immobilized yeast extract (YE) on Cu-dispersed carbon nanofibers (CNFs) to prepare Cu-CNF-YE **(**Figure 3A) 106. YE was capable of converting glucose to ethanol in diabetic wounds, removing high levels of glucose from the wounds while also exerting antibacterial effects through ethanol, and acting synergistically with copper to promote diabetic wound healing.

Combining some bioactive substances to construct nano-biomaterials is a relatively simple method. In this section, we further focus on some nanomaterials such as drug delivery nano-systems, H_2_O_2_ self-supply systems, and nanozymes as they help to realize multifunctional wound treatment strategies.

### Drug delivery nano-systems

Drug delivery nano-systems, which transport and release drugs to specific locations to function, have been used to load bioactive substances and gases to anti-infection and promote wound healing due to their advantages of crossing specific barriers, high loading capacity, slow drug release and high bioavailability 107. Sun et al. developed cinnamaldehyde-loaded nanorods with NIR-mediated drug release properties using a chelate composed of Cu^2+^ and gallic acid (GA). The inherent antibacterial ability of cinnamaldehyde and the antioxidant capacity of GA enabled the nanorods to effectively accelerate the healing of infected wounds 108.

HKUST-1 is a metal-organic framework (MOF) formed by the coordination of 1,3,5 benzenetricarboxylate with copper ions and has a porous structure that has been shown to be useful for loading gas 109. Recently, Zhang et al. prepared nitrogen monoxide (NO)-loaded secondary amine group modified HKUST-1 (NO@HKUST-1) (Figure 3B) 13. NO@HPG was further prepared by blending NO@HKUST-1 particles into polycaprolactone (PCL) (Figure 3C). NO@HKUST-1 was able to release NO by displacing it with water. PCL effectively isolated water and prevented the rapid release of NO from NO@HKUST-1. With the slow degradation of PCL, NO@HKUST-1 came in contact with water and slowly released NO over 14 days. At the wound site, NO@HKUST-1 in NO@HPG released NO and Cu^2+^, both of which synergistically promote angiogenesis and accelerate diabetic wound healing. The Bohr effect refers to the fact that increasing the concentration of CO_2_ can lower the pH and reduce the affinity of hemoglobin for oxygen to release oxygen 110. In a recent study, Li et al. prepared NIR-responsive PEGylated-h-CuS/bicarbonate 111. When the temperature of the material increased, the bicarbonate decomposed to release CO_2_, which increased the local oxygen supply to the wound by lowering the local pH and promoting the release of oxygen from hemoglobin.

### H_2_O_2_ self-supply and Fenton-like reaction system

**Figure 4:**
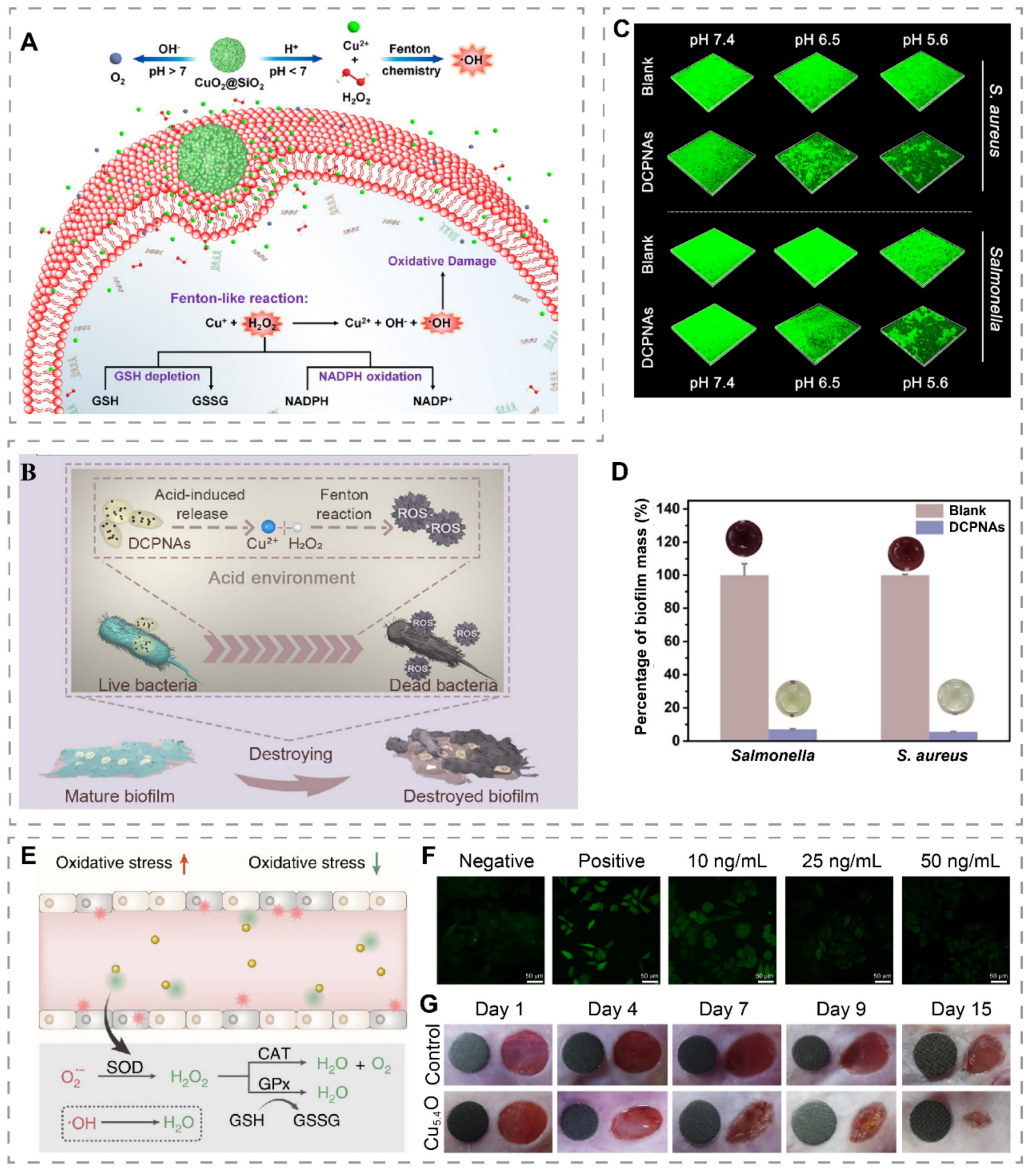
H_2_O_2_ self-supply and Fenton-like reaction systems or nanozyme for wound treatment. (A) pH-responsive CuO_2_@SiO_2_ nanospheres for Fenton-like reaction and O_2_ generation. Reproduced with permission from 39. Copyright 2021, American Chemical Society. (B) Schematic illustration of DCPNAs entering biofilm and antibacterial via acid-induced ROS production. (C) 3D CLSM images of biofilms showing the effect of DCPNAs in inhibiting biofilms under different pH conditions. (D) Effect of DCPNAs in disrupting biofilms (MTT assay). Reproduced with permission from 113. Copyright 2021, American Chemical Society. (E) Mechanism of ROS scavenging by Cu_5.4_O USNPs. (F) ROS staining (green fluorescence) images of HEK293 cells treated with different concentrations of Cu_5.4_O USNPs. (G) Diabetic wounds treated with Cu_5.4_O USNPs at different time points (The diameter of the green disc is 6 mm). Reproduced with permission from 67. Copyright 2020, Springer Nature.

Although copper has a strong Fenton-like reaction capacity to catalyze the production of ·OH, ROS inherently has a short diffusion distance (~ 20 nm) and lifetime (< 200 ns), and thus antibacterial systems based on Fenton-like reactions should have sufficient H_2_O_2_ substrate to ensure sustained ·OH production 112. Recently, it has been reported that CuO_2_ nanodots can decompose into Cu^2+^ and H_2_O_2_ under acidic conditions, and thus can be used to construct H_2_O_2_ self-supply systems 66. Based on this, Zhang et al. synthesized CuO_2_ nanodots in the presence of PVP and NaOH 40. The obtained CuO_2_ nanodots exhibited good dispersion in water due to their small size and PVP coating and were made into a spray for application on infected diabetic wounds. In the weakly acidic microenvironment of infection, CuO_2_ decomposed to Cu^2+^ and H_2_O_2_, which subsequently triggered Fenton-like reactions to produce the more ·OH, thus effectively killing *Staphylococcus aureus (S. aureus)*, *Escherichia coli (E. coli)*,* Pseudomonas aeruginosa (P. aeruginosa),* and methicillin-resistant *Staphylococcus aureus* (MRSA). In addition, the released Cu^2+^ promotes migration and angiogenesis of human umbilical vein endothelial cells (HUVECs), therefore promoting the healing of infected wounds. In another study, CuO_2_ was encapsulated into mesoporous silica nanoshells to obtain pomegranate-like CuO_2_@SiO_2_ nanospheres for *S. aureus*-infected wound treatment (Figure 4A) 39. The protection provided by the silica nanoshells slowed down the degradation of CuO_2_ under acidic and neutral conditions, allowing CuO_2_ to exert its functionality for a longer period of time. CuO_2_@SiO_2_ exhibited different reaction characteristics at different pH ranges. Specifically, at pH 7.4, CuO_2_@SiO_2_ can act as a powerful oxygen generator. Under acidic conditions, it generated ·OH and depleted bacterial endogenous antioxidants GSH and reduced β-nicotinamide adenine dinucleotide phosphate (NADPH) to assist in the clearance of wound bacteria.

Biofilms are microbial aggregates embedded in extracellular polymeric substances (EPS) matrix consisting of polysaccharides, lipids, proteins, and nucleic acids, which can resist the effects of antimicrobial agents by slowing down bacterial growth and utilizing the EPS barrier 114. EPS matrix delays the penetration of ROS into the biofilm, resulting in insufficient ROS concentration in the biofilm and weakening its antibacterial effect. To address the low level of ROS within the biofilm, Li et al. designed dextran-coated CuO_2_ nanoaggregates (DCPNAs) 113. The dextran on the surface enhanced the diffusion ability of DCPNAs in the biofilm and helped transport CuO_2_ into the biofilm for H_2_O_2_ self-supply and Fenton-like reactions within the biofilm (Figure 4B). DCPNAs showed pH-dependent capability for antibacterial and biofilm inhibition. After 24 hours of co-culture with bacteria, the mass of biofilm formed by DCPNAs-treated *S. aureus* and *Salmonella* at pH 5.6 was less than 10% of that of the PBS-treated group (Figure 4C-D). In *S. aureus*-infected wounds, DCPNAs treatment reduced the number of bacteria in the wound and accelerated wound healing.

### Copper-incorporated nanozymes

Nanozymes are characterized by low cost, good stability, and adjustable enzyme-mimetic activity, and are widely used for antibacterial, biosensors and disease treatments 115-117. The main strategy of treating wounds with copper-containing nanozymes is to generate ROS through POD and OXD-like activities, and use the generated ROS to promote wound healing via antibacterial and anti-biofilm formation 118, 119. At present, copper-containing nanozymes have been applied to wound treatment (Table 1).

Killing bacteria by ROS production catalyzed by nanozymes is a commonly used therapeutic strategy. Recently, Li et al. synthesized a cuprous oxide/platinum (Cu_2_O/Pt) nanocubes with POD, CAT, and OXD-like activities for infected wound treatment 120. By co-application with exogenous H_2_O_2_, H_2_O_2_ can be converted into ·OH and O_2_^•-^, thus exerting a potent antibacterial effect. The high levels of glucose in diabetic wounds induce high levels of ROS and reasons oxidative stress, which is an important cause of difficult wound healing in diabetic patients 123. ROS scavenging by nanozymes is an effective strategy to improve the microenvironment of diabetic wounds. Recently, Liu et al. constructed ultra-small Cu_5.4_O NPs (Cu_5.4_O USNPs) with CAT, SOD, and GPx-like activities, whose SOD-like activity was about 21% of the natural SOD activity and can convert O_2_^•-^ to H_2_O_2_, which was further scavenged by GPx-like activity or converted to O_2_ by CAT-like activity **(**Figure 4E) 67. In vitro, Cu_5.4_O USNPs showed a dose-dependent ROS scavenging capacity (Figure 4F). In vivo study found faster wound healing in diabetic mice treated with Cu_5.4_O USNPs (Figure 4G).

Although local hypoxia in the wound is a contributing factor to angiogenesis, this is limited to acute hypoxia 124, 125. On the contrary, chronic local hypoxia will affect the metabolic capacity of cells and hinder the generation of neovascularization and wound healing 124, 125. It has been shown that oxygen pressure greater than 50 mmHg is beneficial for wound healing 126. Recently, Xi et al. designed copper adhered carbon spheres (Cu-HCSs) with CAT, POD, and SOD-like activities and were able to catalyze H_2_O_2_ to provide O_2_ to the wound via CAT-like activity 12. Co-application of Cu-HCSs with H_2_O_2_ to *S. aureus*-infected wounds resulted in significantly faster healing of the wounds. Liu et al. prepared copper-doped carbon dots (Cu-CDs) with POD and CAT-like activities 121. 240 µg/mL Cu-CDs were able to catalyze the generation of more than 30 ppm O_2_ from 4 mM H_2_O_2_. Cu-CDs were used on wounds infected with *S. aureus* alone or with mixed bacteria and were found to reduce inflammatory exudation and significantly promote wound healing.

## Copper-doped bioactive glasses

**Figure 5:**
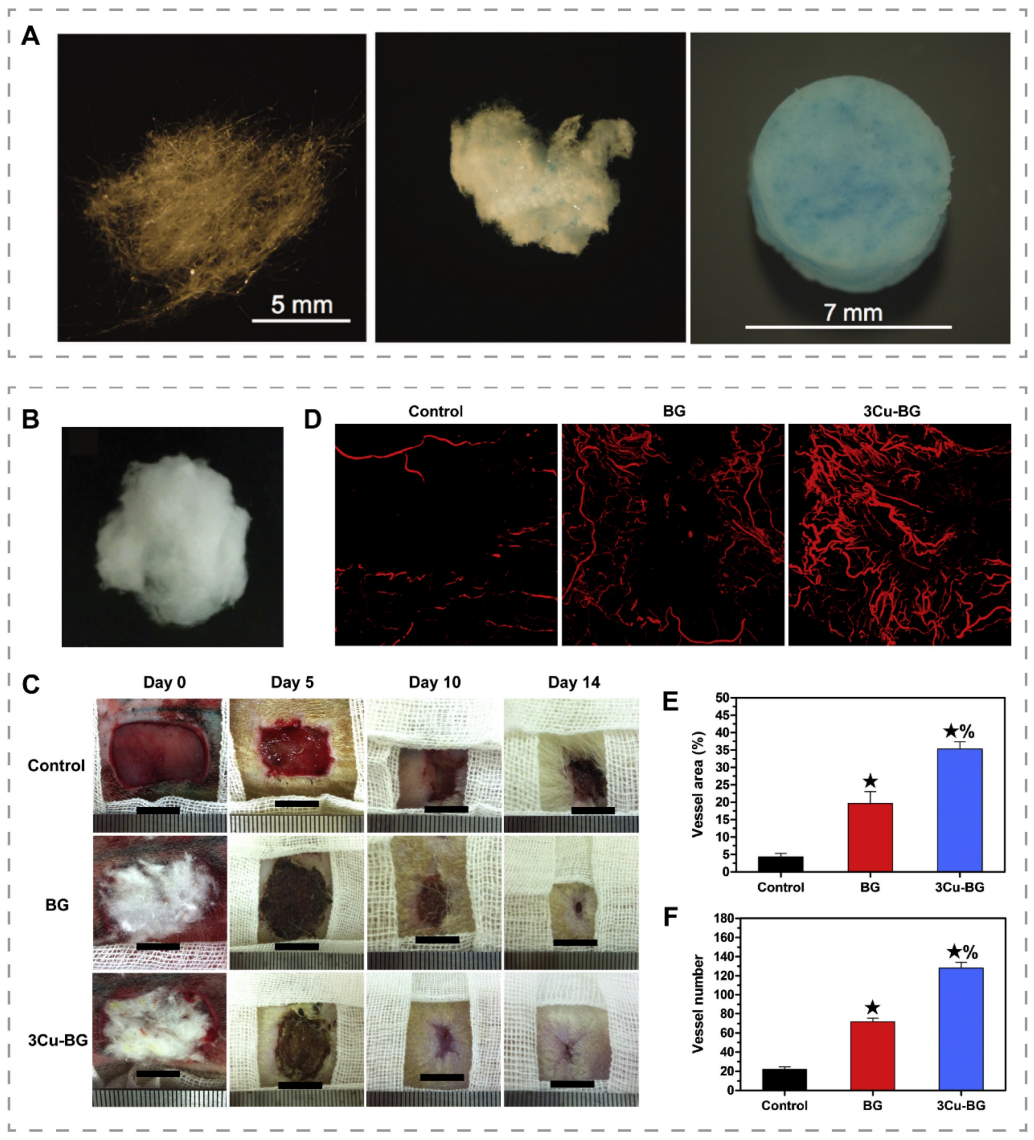
Bioactive glass microfibers for promoting angiogenesis and wound healing. (A) Images of 13-93B3 bioglass (left), 13-93B3Cu bioglass fiber (middle) and mat of 13-93B3Cu bioglass fiber (right). Reproduced with permission from 132. Copyright 2014, Wiley-VCH. (B) Photographs of the bioactive glass dressings containing 3.0 wt% CuO. (C) Representative images of wounds at different time points. (D) Micro-CT evaluation of blood vessels at the wound site. Quantitative analysis of vessel area (E) and vessel number (F). ★, compared to the control, p < 0.05; %, compared to BG, p < 0.05. Reproduced with permission from 16. Copyright 2015, Elsevier.

Bioactive glass was first manufactured in the late 1960s and is widely used in medical research 127. Bioactive glasses have been found to inhibit inflammation and promote angiogenesis and re-epithelialization, which is related to the released calcium, silicon, and phosphorus ions 128-130. By changing the composition of bioactive glass, it is possible to adjust its degradation rate and alter its bioactivity, allowing bioactive glass to be considered for soft tissue repair 131. Recently, Lin et al. added 0.4 wt% CuO to 13-93B3 glass microfibers and obtained 13-93B3Cu glass microfibers with enhanced angiogenesis **(**Figure 5A) 132. Zhao et al. prepared a cotton-like wound dressing consisting of borate bioactive glass microfibers with an average diameter of 0.85 µm by blowing high-pressure gas onto molten glass and then quenching the fibrous material (Figure 5B) 16. The dressings were applied to full-thickness skin wounds of rats and found that the bioactive glass dressings containing 3.0 wt% CuO (3Cu-BG) treated wounds formed more blood vessels and closed faster than wounds treated with bioactive glass without copper (Figure 5C-F). To address the inflammation caused by the sharp morphology of glass particles, Li et al. constructed copper-containing bioactive glass coating on the surface of the natural eggshell membrane (ESM) by using pulsed laser deposition technique 133. The coating on ESM showed enhanced angiogenesis and tissue repair.

Bioactive glasses can mainly be prepared by melting or sol-gel methods 134. In 2004, Yan et al. prepared mesoporous bioactive glass (MBG) for the first time by using surfactant templating and sol-gel methods 135. Recently, copper-doped MBG has demonstrated enhanced angiogenesis and extracellular matrix production 136. With adjustable pore size, high pore volume and high specific surface area, MBG is expected to serve in the future as a delivery platform for copper, other metal ions, drugs or cytokines to enhance tissue repair 137.

## Copper-incorporated polymer scaffolds

In recent years, polymers have been widely used in the study of wound healing due to their degradability, ability to absorb water and moisture, and appropriate mechanical properties, permeability, and porosity 138. For some large and deep wounds, scaffolds can mimic the extracellular matrix structure, provide mechanical support for cells, and guide cell adhesion, proliferation, migration, and differentiation to help wound healing 139. Polymer scaffolds have been criticized for their limited ability to absorb water. However, recent study has indicated that by adjusting the composition and structure of the polymer, polymer dressings with a wettability gradient enable unidirectional transport of fluids for effective removal of wound exudates 140. Undoubtedly, this makes polymer dressings more suitable for the needs of wound treatment.

The addition of copper not only provides the polymeric scaffold with antibacterial ability, but also changes the mechanical properties of the polymeric scaffold 141. Recently, Al-Saeedi prepared cellulose acetate (CA) fiber scaffolds containing CuO (CuO@CA) by electrospinning technology and found that the addition of CuO not only conferred the antibacterial ability to cellulose acetate but also dose-dependently increased hydrophilicity and tensile strength, making it more suitable for wound dressing 142. Jaganathan et al. found that CuSO_4_ increased the hydrophilicity and tensile strength of polyurethane (PU) fibers 143. While in another study, it was found that the moisture vapor transmission rate (MVTR) and air permeability (AP) of polyvinyl alcohol (PVA) scaffolds decreased with increasing CuO content, because CuO NPs blocked the pores between nanofibers 144.

Currently, a range of copper-doped polymer scaffolds have been developed for wound repair 10, 48. Through the release of copper ions from polymer scaffolds, copper-containing polymer scaffolds have demonstrated more angiogenesis, matrix deposition and faster wound healing in animal studies 10, 48. It is worth noting that the drug release from polymeric scaffolds is usually continuous and uncontrollable 142. In contrast, wound microenvironment-responsive polymeric scaffolds can achieve selective drug release, improve drug utilization, and better promote wound healing 145. Recently, Darder et al. prepared a blue cellulose/PAH-CuHARS foam scaffold, a cellulose scaffold containing copper-cystine high ratio structure (CuHARS) 17. The CuHARS in the scaffold not only slowly degraded to release Cu^2+^ but also catalyzed the production of NO upon contact with the blood component S-nitrosocysteine (CysNO), which has the potential to exert antibacterial, anti-inflammatory, anticoagulant and wound healing promoting effects. Since HKUST-1 is prone to decomposition under acidic conditions, a recent study has prepared HKUST-1/chitosan (HKUST-1/CS) films 146. The HKUST-1/CS films can release more Cu^2+^ to antibacterial under the stimulation of low pH and promote infected wound healing.

## Copper-incorporated hydrogels

Hydrogels are cross-linking networks formed by hydrophilic polymers, which are characterized by their ability to absorb liquid several times their own volume 147. Natural hydrophilic polymers used to synthesize hydrogels include hyaluronic acid (HA), alginate, chitosan, collagen, gelatin, starch, and other biodegradable natural polymers 148, 149. In recent years, synthetic polymers such as polyacrylamide (PAM), polyethylene glycol (PEG), and polymethyl methacrylate (PMMA) have also been used to develop hydrogels due to their low immunogenicity and better mechanical properties 148. With an appropriate swelling ratio and porous structure, hydrogel allows the exchange of nutrients and gases and plays the role of absorbing exudate and moisturizing in wounds 150. In addition, through the modification of the molecular network of hydrogels or the modulation of the components, some hydrogels can also possess self-healing and adhesion properties, which can help to achieve physical hemostasis and provide an appropriate protective barrier for wounds 151. Particularly after the introduction of copper, the antibacterial properties of hydrogels can be significantly enhanced. Therefore, copper- incorporated hydrogels have great potential for wound treatment.

**Figure 6:**
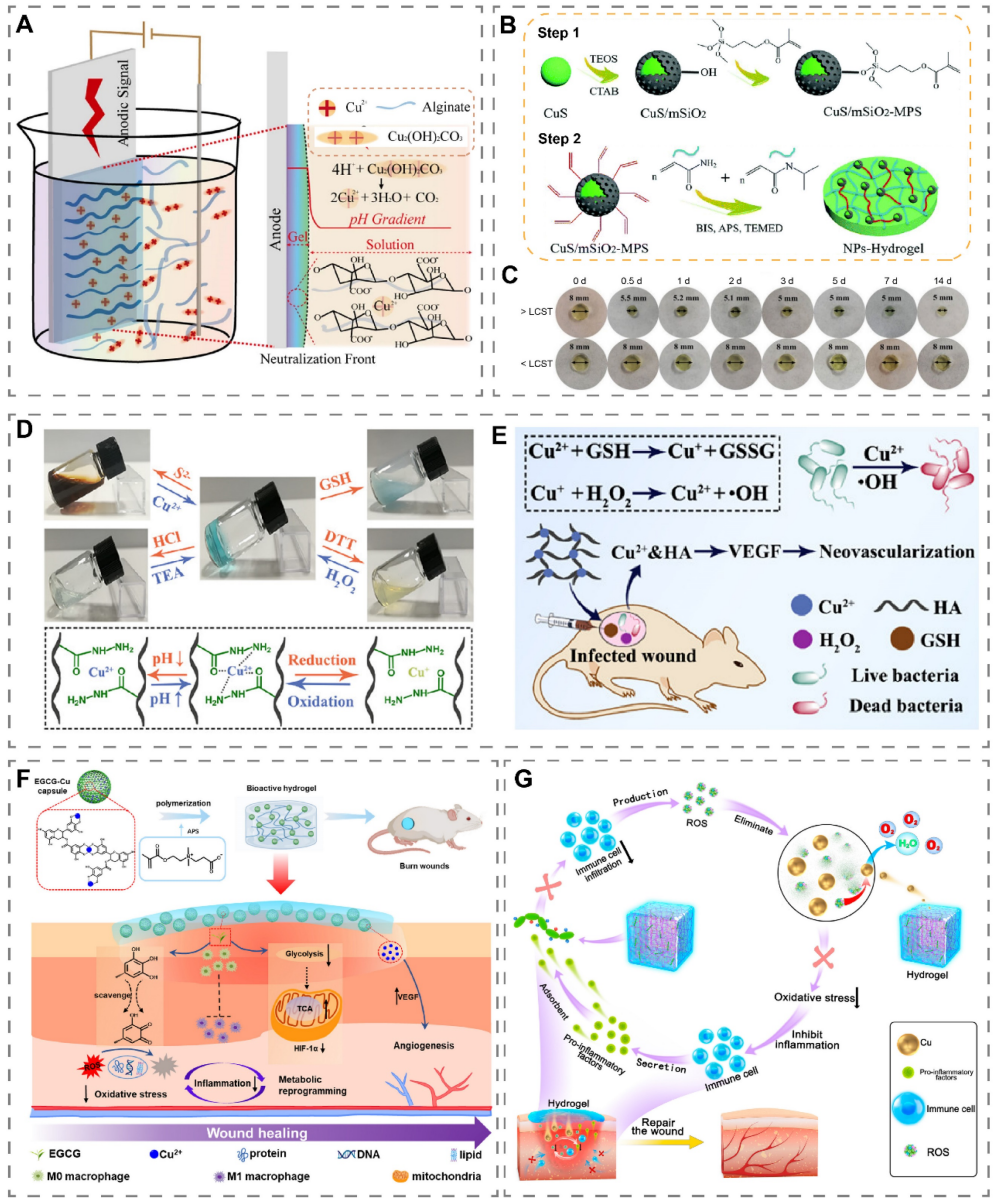
Preparation and application of copper-containing hydrogels. (A) Mechanism of preparing Cu^2+^-Alg hydrogels by electrodeposition. Reproduced with permission from 18. Copyright 2021, Springer Nature. (B) Schematic diagram of the preparation of CuS/mSiO_2_-MPS/poly(NIAAm-co-AAm) hydrogel. (C) Temperature-induced volume change of hydrogel. >LCST (37 °C), <LCST (28 °C). Reproduced with permission from 154. Copyright 2018, Royal Society of Chemistry. (D) Reversible sol-gel phase transition of HA-Cu hydrogel initiated by S^2-^/Cu^2+^, pH, and redox. (E) Schematic illustration of HA-Cu hydrogels for antibacterial and wound healing promotion. Reproduced with permission from 155. Copyright 2022, American Chemical Society. (F) Schematic diagram of the preparation and wound healing mechanism of EGCG-Cu@CB hydrogel. Reproduced with permission from 156. Copyright 2023, Elsevier. (G) Schematic diagram of Cu_5.4_O@Hep-PEG hydrogel accelerating wound healing through ROS clearance and pro-inflammatory factor capture. Reproduced with permission from 157. Copyright 2021, Elsevier.

Generally, two main methods are used to prepare copper-containing hydrogels, one is to mix copper with the hydrogel prepolymer and then cross-link it, and the other is to dry the cross-linked hydrogel and then soak the copper-containing liquid to make copper enter the pores of the hydrogel 152, 153. Recently, a Cu^2+^ cross-linked alginate hydrogel (Cu^2+^-Alg) was prepared by the electrodeposition method (Figure 6A) 18. Specifically, a three-electrode system was placed in an alginate liquid containing the insoluble salt Cu_2_(OH)_2_CO_3_. After the application of a transient current, Cu^2+^ was released from Cu_2_(OH)_2_CO_3_ and cross-linked with alginate to obtain a Cu^2+^-Alg hydrogel on the anode surface. Compared with the conventional method, this method has the advantages of high porosity (more than 50%), fast preparation, uniform copper distribution and adjustable hydrogel thickness and copper content.

### Stimuli-responsive hydrogels

For most hydrogels, slow release of Cu^2+^ to antibacterial, modulate inflammation, promote angiogenesis, collagen deposition, matrix remodeling and re-epithelialization are their main strategies to accelerate wound healing 158, 159. In recent years, stimuli-responsive hydrogels have received widespread attention because of their ability to control the release of drugs in response to changes in the wound microenvironment 160. Alginate hydrogels are bacterial and pH-responsive and have been used to prepare copper-containing hydrogels to accelerate tissue repair 161, 162. Shahriari et al. prepared a pH-responsive Cu^2+^ cross-linked sodium alginate hydrogel 163. Alginate hydrogel was hydrolyzed under acidic conditions in wounds, releasing more copper ions to resist infection. The bacteria-responsive property of alginate occurrs as a result of alginate lyase produced by bacteria which degrades alginate through the β-elimination of glycosidic bonds 164. Chen et al. constructed alginate microcapsules containing both Cu-MOFs and Zn-MOFs using microfluidic electrospray method 165. The microcapsules accelerated degradation with increasing *E. coli* concentration and released Cu^2+^ and Zn^2+^ to activate copper/zinc superoxide dismutase (Cu/Zn-SOD) to scavenge the accumulated ROS in the infected wound.

Temperature-responsive hydrogels undergo a sol-gel phase transition at the appropriate temperature and are suitable copper-loaded platforms 160. Normally the human skin temperature is 26 °C-34 °C 166. Wang et al. obtained PHN-Cu solutions with a lower critical solution temperature (LCST) of 29 °C by direct doping of Cu^2+^ in poly-(HEMA-co-NIPAM) solution 167. PHN-Cu underwent a rapid phase change when applied to wounds, with the potential to stop bleeding by increasing wound pressure and to release Cu^2+^ to accelerate wound healing in diabetic rats. Recently, Tao et al. have demonstrated that the photothermal effect can elevate the temperature of copper-containing hydrogels, thereby accelerating the release of Cu^2+^ 168. Li et al. added mesoporous silica (mSiO_2_) coated CuS NPs to hydrogels composed of acrylamide (AAm) and *N*-isopropylacrylamide (NIPAAm) to obtain CuS/mSiO_2_-MPS/poly(NIAAm-co-AAm) hydrogel (Figure 6B) 154. At 37 °C (>LCST), the hydrogel diameter shrank from 8 mm at the beginning to 5 mm on the third day (Figure 6C). Based on this, NIR light irradiation can reduce the volume of the hydrogel by heating through the photothermal effect of CuS, thus releasing more Cu^2+^.

Recently, Qian et al. prepared an HA-based hydrogel capable of responding to balanced ion pairs (S^2-^/Cu^2+^), pH, and redox (Figure 6D) 155. Notably, when Na_2_S was added to the HA-Cu hydrogel, Cu^2+^ in the hydrazide-Cu coordination unit combined with S^2-^ to form photothermal agent CuS, which enabled the HA-Cu hydrogel to possess photothermal properties. Combining HA-Cu hydrogel with GSH effectively accelerated infected wound healing through ·OH and the release of Cu^2+^ (Figure 6E).

### Hydrogels for modulating the wound microenvironment

Glucose oxidase (GOD) catalyzes the oxidation of glucose in the wound bed, decreasing the wound glucose concentration, and is thought to be useful in constructing cascade reaction systems. Recently, Wang et al. developed a hydrogel microparticle integrating GOD, CuO_2_, pH-responsive acrylic acid, and hyaluronic acid methacryloyl for wound healing and real-time monitoring of diabetic wounds 169. GOD was able to convert glucose in the wound bed to H_2_O_2_ and gluconic acid, while lowering the pH of the wound and accelerating the breakdown of CuO_2_. Notably, both the glucose-catalyzed product and the CuO_2_ decomposition product contained H_2_O_2_, which could be used in copper-mediated Fenton-like reactions, resulting in a highly effective antibacterial effect. Moreover, the microparticle was pH-responsive, with a spectral blue-shift trend at decreasing pH, which could visualize the pH of the wound and be used to assess the glucose-catalyzed process and the wound microenvironment.

The immune microenvironment of wounds is susceptible to over-activation by interference from factors such as infection, which leads to a persistent inflammatory state and high oxidative stress levels at the wound site. The construction of copper-containing hydrogels with anti-inflammatory, antibacterial and antioxidant properties is expected to correct the pathologies that lead to delayed wound healing 65. Yang et al. prepared a multifunctional hydrogel based on decellularized pomelo peel (DPP) coated with PVA-TSPBA hydrogel and doped with antibacterial gallic acid/copper MOFs 68. DPP had good anti-inflammatory and antioxidant properties, and PVA-TSPBA hydrogel, as a ROS-sensitive hydrogel, could achieve ROS scavenging in wounds. The hydrogel stimulated M2 macrophage polarization and adjusted the wound microenvironment from a pro-inflammatory state to a pro-regenerative state, thereby effectively promoting the healing of infected wounds and reducing scar formation. It has been reported that excessive ROS in the wound bed might lead to mitochondrial dysfunction and enhanced glycolytic metabolism in macrophages, which induces macrophage M1-type polarization 170. Enhanced glycolytic metabolism in M1-type macrophages leads to the accumulation of succinate by affecting mitochondrial oxidative phosphorylation and the tricarboxylic acid cycle, which in turn results in mitochondrial ROS production and initiates the uncontrolled inflammatory cycle. Li et al. prepared a hydrogel (EGCG-Cu@CB) encapsulating epigallocatechin gallate (EGCG) and Cu^2+^ (Figure 6F) 156. EGCG not only scavenged excessive ROS in the wound bed but also metabolically reprogrammed macrophages by inhibiting glycolysis and normalizing the TCA cycle, which ultimately restored the redox homeostasis and blocked pro-inflammatory signaling. The accumulation of ROS and pro-inflammatory factors in wounds can further amplify local inflammation through inflammatory circulation. Current studies usually focus more on clearing ROS from wounds, ignoring the harm of pro-inflammatory factors already present in wounds 21, 67. Recently, Peng et al. proposed a 'weeding and uprooting' treatment strategy for dual clearance of ROS and inflammatory factors from wounds (Figure 6G) 157. In this study, heparin-PEG hydrogels loaded with Cu_5.4_O USNPs (Cu_5.4_O@Hep-PEG) were prepared. Wherein, the Cu_5.4_O nanozyme can clear excess ROS, and heparin-PEG hydrogels capture monocyte chemoattractant protein-1 (MCP-1) and IL-8 through electrostatic interaction, thus synergistically reducing macrophage and neutrophil infiltration, lowering inflammation level, and accelerating diabetic wound healing.

## Copper-containing photothermal nanomaterials

PTT has received much attention in medical research as a non-contact, modifiable treatment modality 54. The copper chalcogenide Cu_2-x_E (E = S, Te, Se, 0 ≤ x ≤ 1) based materials have good NIR-I and NIR-II absorption properties due to the localized surface plasmon resonance (LSPR) arising from copper defects, which results in the oscillating electromagnetic field of the driving light interacting with the Cu_2-x_E free carriers 171. Among the many copper-based photothermal agents, CuS has been most widely used in wound repair due to its low cost, low toxicity, simple preparation, and excellent photothermal conversion efficiency 172. Current applications of copper-based photothermal materials in wounds include antibacterial and promotion of wound healing. The antibacterial activity is mainly related to the heat-induced killing effect by PTT and the release of Cu^2+^ 54. Notably, the photothermal effect can also be used to modulate drug release, which is non-contact and active 173. It will contribute to on-demand drug delivery in wounds, which is highly promising in wound therapy. Recently, a series of targeted antibacterial strategies and synergistic therapies based on copper-containing photothermal materials have been proposed for wound treatment (Table 2).

### Targeted photothermal antibacterial materials

**Figure 7:**
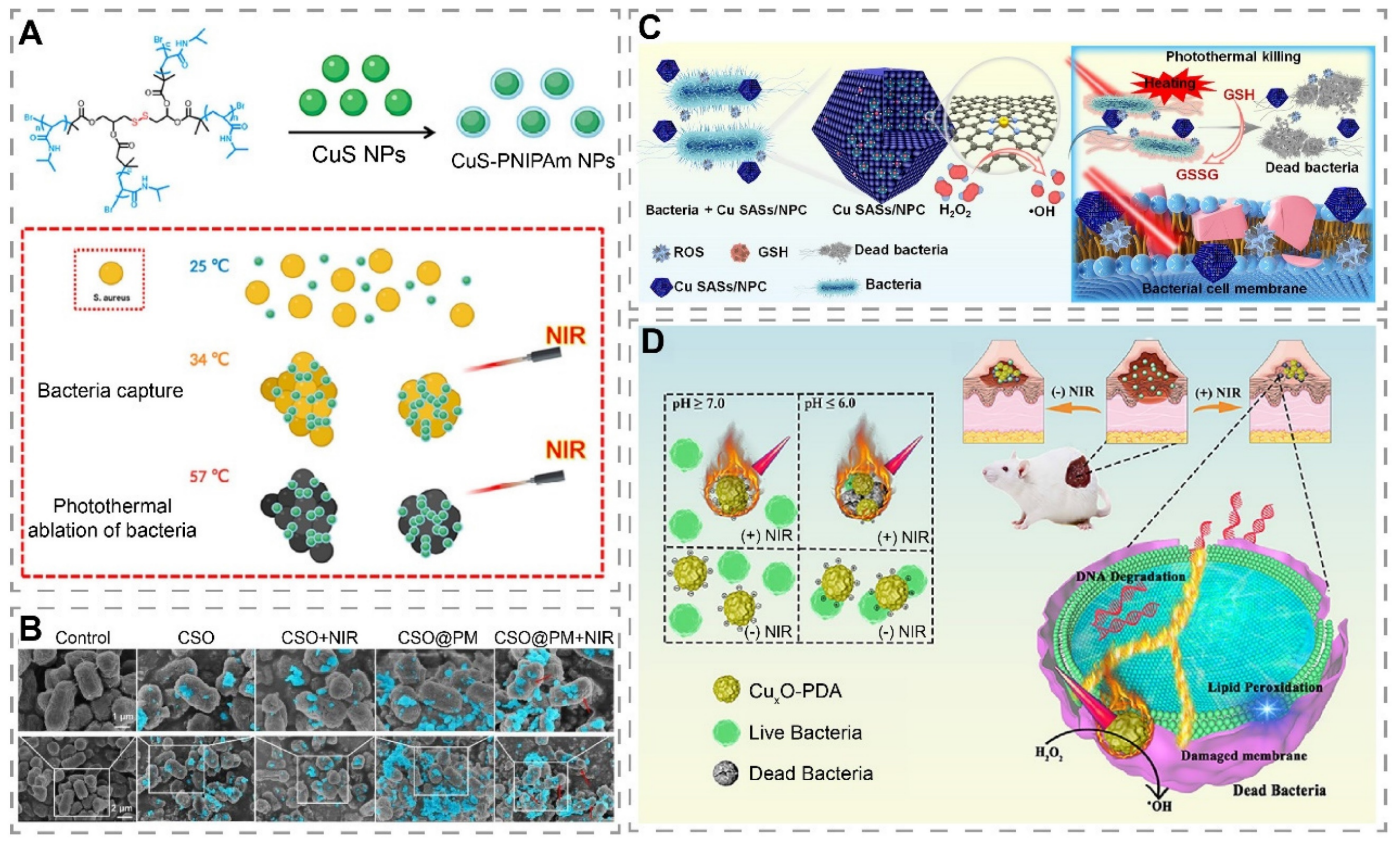
Targeted photothermal antibacterial materials and photothermal catalytic materials for wound treatment. (A) Schematic representation of the preparation and antibacterial mechanism of CuS-PNIPAm NPs. Reproduced with permission from 15. Copyright 2022, Oxford University Press. (B) SEM images of *P. aeruginosa* treated with PBS, CSO, CSO@PM, CSO + NIR, CSO@PM + NIR. Blue indicates NPs. Scale bar: 1 µm (top), 2 µm (bottom). Reproduced with permission from 175. Copyright 2021, Springer Nature. (C) Schematic diagram of Cu-SASs/NPC for the treatment of infected wounds through photothermal conversion and induction of bacterial oxidative stress imbalance. Reproduced with permission from 49. Copyright 2021, Elsevier. (D) Schematic illustration of Cu_x_O-PDA antibacterial and wound applications. Reproduced with permission from 178. Copyright 2022, American Chemical Society.

Although photothermal materials can kill bacteria by increasing temperature, this excess in heat can potentiate damage to the normal surrounding tissues 184. For wounds, after the normal skin cells around photothermal materials are killed by excessive temperature, wound healing can only be achieved by the migration of cells from more distant healthy tissue into the wound, which means that the wound healing may be delayed 182. To enhance the targeting killing effect of photothermal materials on bacteria, Dai et al. constructed poly(5-(2-ethyl acrylate)-4-methylthiazole-g-butyl) (PATA-C4) modified CuS NPs (PATA-C4@CuS) for the precise killing of bacteria through electrostatic mediated bacterial membrane targeting 174. PATA-C4@CuS was positively charged due to the presence of quaternary ammonium groups, and can bind to negatively charged bacterial membranes by electrostatic interaction to form a material-bacterial conjugate precipitate and kill bacteria via PTT and PDT. In contrast, PATA-C4@CuS combined with NIR showed good biocompatibility with fibroblasts, which further confirmed the bacterial targeting effect of PATA-C4@CuS. The difference in toxicity of PATA-C4@CuS to bacteria and fibroblasts could be attributed to the point that bacterial membranes hold more negative surface charges. In another study, Wang et al. increased the hydrophobicity of the four-armed poly(N-isopropylacrylamide) (PNIPAM) on the surface of CuS-PNIPAM NPs through photothermal effect, which enhanced the adhesion to bacterial membranes for bacterial capture and elimination (Figure 7A) 15. Interestingly, Cu^2+^ released from the nanoparticles can subsequently chelate with the amide group of the PNIPAM shell, avoiding the local accumulation of Cu^2+^. Peng et al. prepared a platelet membrane (PM)-encapsulated mesoporous copper silicate microspheres (CSO) (CSO@PM) that targeted bacteria via Toll-like receptors, formyl peptide receptors, and chemokine receptors of PM (Figure 7B) 175. In vivo studies have shown that CSO@PM + NIR can reduce wound inflammation and accelerate wound healing.

### Photothermal catalytic materials

It has been shown that photothermal catalytic materials, which generated ·OH by POD-like activity, can exert stronger antibacterial effects in synergy with the heat-induced killing effect of PTT 185. Wang et al. designed Cu single atomic site/N-doped porous carbon photothermal catalytic antibacterial nanoplatforms (Cu SASs/NPC) for the treatment of infected wounds (Figure 7C) 49. Cu SASs/NPC had POD-like and GPx-like activities that catalyzed the production of ·OH and depletion of GSH, respectively. Meanwhile, Cu SASs/NPC showed a photothermal conversion efficiency of 82.78% under NIR light (808 nm, 1.0 W/cm^2^) irradiation. In vitro, Cu SASs/NPC+H_2_O_2_+NIR resulted in high ROS production in bacteria via POD-like activity and PTT. As GSH was depleted, ROS was able to exert better antibacterial effects, as shown by killing almost all *E. coli* and MRSA. In vivo, it showed the least number of residual bacteria, the least inflammatory response, and the fastest healing rate in MRSA-infected wounds. In another study, Bai et al. prepared an ultrathin two-dimensional N-doped porous carbon nanosheet supported Cu single atoms (CuN_x_-CNS, x = 2 or 4) with tunable N-coordination structures, which exhibited excellent POD, OXD, and CAT activities as well as good photothermal performances in the NIR-II window 186. Compared with CuN_2_-CNS, CuN_4_-CNS possessed a stronger electron-pushing effect and oxygen adsorption ability, and thus exhibited stronger CAT and OXD activities, which significantly enhanced ROS generation. Notably, the photothermal effect under NIR-II irradiation could further promote ROS generation. The excellent enzyme mimetic activity synergized with the photothermal effect to effectively kill bacteria and eliminate biofilm. Due to the multiple facilitating effects of copper in wound healing, CuN_4_-CNS could reduce the inflammatory response of infected wounds and promote superficial wound healing. Encouragingly, CuN_4_-CNS also showed good therapeutic effects on deep implant-related infections, effectively reducing inflammation.

Considering the characteristics of short lifetimes and short diffusion distance of ROS, building a targeted bacterial system helps to produce toxic ·OH at a short distance, thereby improving the antibacterial efficacy 187. He et al. modified Cu_x_O with polydopamine (PDA) to construct a material with both photothermal and catalytic properties (Cu_x_O-PDA), where PDA was used as a photothermal agent and a bacterial target binding site (electrostatic interaction) and Cu_x_O was used to mimic POD activity (Figure 7D) 178. In vitro study showed that the optimal temperature for POD-like activity of Cu_x_O-PDA was 55 °C. Under NIR light irradiation, the elevated temperature helped Cu_x_O-PDA to produce more ·OH. In vitro, Cu_x_O-PDA+H_2_O_2_+NIR displayed a stronger bacterial killing effect than Cu_x_O-PDA+H_2_O_2_ and Cu_x_O-PDA+NIR, suggesting a potential synergistic antibacterial effect of PTT and POD-like activity of Cu_x_O-PDA under NIR light irradiation.

### Low-temperature PTT

Local temperatures above 45 °C can reason necrosis of normal skin cells and induce local inflammation 188. The copper-containing photothermal materials currently applied to wounds can result in wounds above 50 °C, with the risk of skin burns 189. Recently, low-temperature PTT has received a lot of attention in wound healing due to its mild thermal stimulation that can be antibacterial, promote angiogenesis, and has good biosafety 190, 191. However, the antibacterial capacity of low-temperature PTT alone is limited, thus low-temperature PTT combined with PDT, antibacterial drugs, or gas therapy to enhance the antibacterial capacity, are promising strategies for the treatment of infected wounds 192.

**Figure 8:**
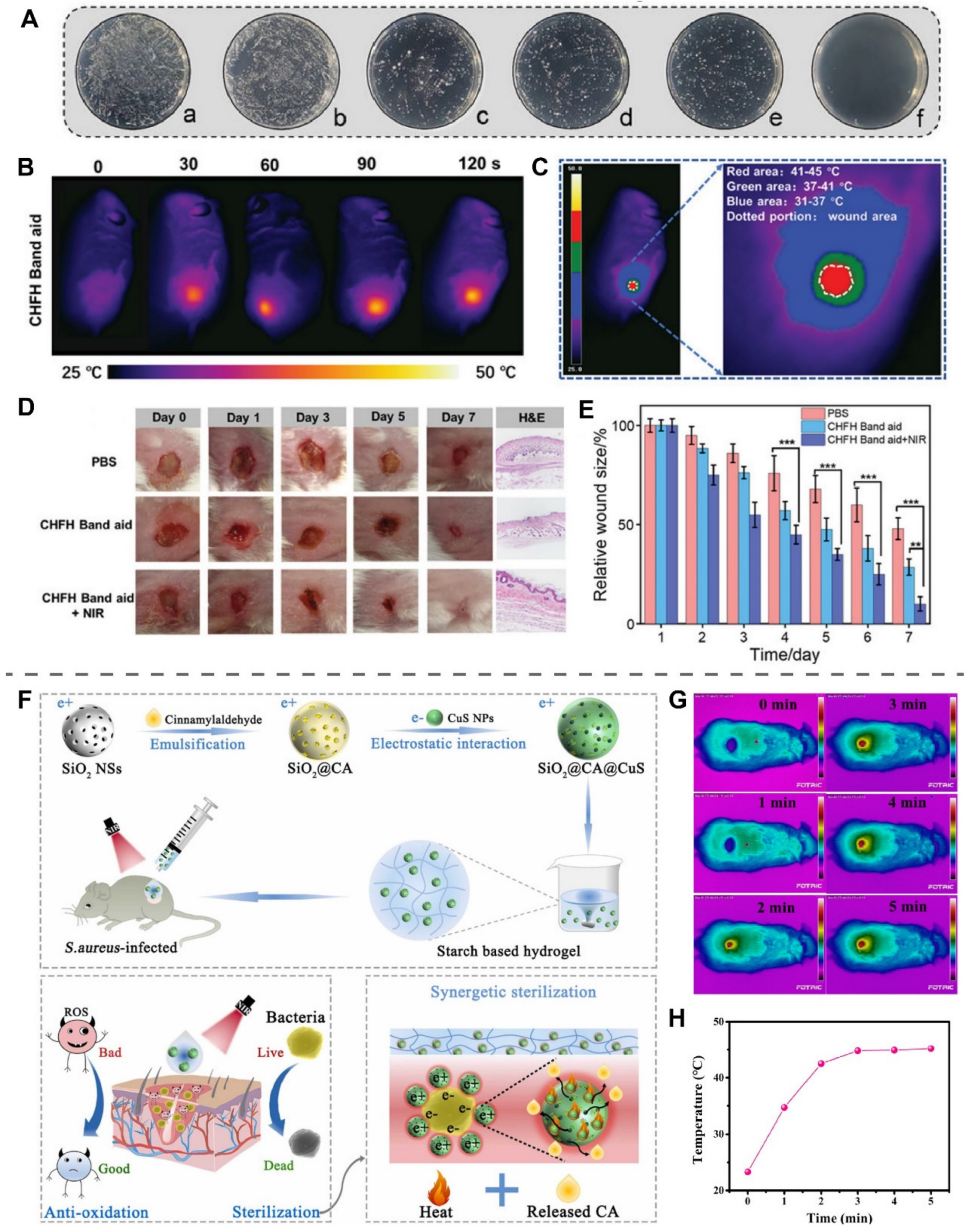
Low-Temperature PTT of copper-based photothermal materials. (A) Antibacterial effect of CHFH on *S. aureus*. (a: PBS, b: NIR, c: HFH, d: CHFH, e: CuSNPs + NIR, f: CHFH + NIR.) (B) Local temperature of the wound at different time points after NIR irradiation. (C) Temperature distribution of the wound after NIR irradiation. (D) Photographs of *S. aureus*-infected wounds at different time points after treatment. (E) Statistical analysis of relative wound size. Reproduced with permission from 180. Copyright 2021, Wiley-VCH. (F) Schematic illustration of antibacterial mechanism of SiO_2_@CIN@CuS. (G) The photothermal effect of SiO_2_@CIN@CuS in *S. aureus* infected wounds under NIR irradiation (980 nm, 1 W/cm^2^, 5 min) (CIN in the text and CA in this figure both represent cinnamaldehyde). (H) The temperature of wounds treated with SiO_2_@CIN@CuS starch hydrogel after NIR irradiation. Reproduced with permission from 14. Copyright 2022, Elsevier.

Lin et al. prepared an HA hydrogel loaded with CuS NPs using Fe^3+^-EDTA complexes as cross-linking agents (CHFH, CuSNPs-HA-Fe^3+^-EDTA hydrogel) 180. NIR light irradiation of CHFH exerted a potent antibacterial effect through mild PTT (45 °C) of CuS in synergy with chemodynamic therapy of Fe^3+^ (Figure 8A). CHFH was prepared as a wound patch placed on the *S. aureus*-infected wounds, and NIR light (808 nm, 0.5 W/cm^2^) irradiation for 2 min was able to control the temperature of the wounds at 45 °C (Figure 8B-C). The wounds treated with CHFH+NIR light irradiation were completely healed on the seventh day, faster than the group using CHFH band aid without NIR light irradiation (Figure 8D-E). Further culture of the bacteria from the wounds found that CHFH+NIR showed the strongest antibacterial ability with an antibacterial efficiency of 98%.

Recently, Li et al. used SiO_2_ as a carrier loaded with cinnamaldehyde (CIN) and CuS NPs to obtain SiO_2_@CIN@CuS, which had an electrostatic binding effect on bacteria, for the treatment of *S. aureus*-infected wounds (Figure 8F) 14. CuS exerted antioxidant effects and promoted the release of CIN by increasing the temperature of SiO_2_@CIN@CuS through photothermal conversion. Payable to the synergistic effect of CIN and PTT, SiO_2_@CIN@CuS exhibited more than 99% resistance to *S. aureus* and *E. coli* under NIR light irradiation at 43 °C in vitro. In the *S. aureus*-infected wound, starch hydrogel loaded with SiO_2_@CIN@CuS was irradiated with NIR light (980 nm, 1.0 W/cm^2^, 5 min), and the wound temperature reached a peak of 45 °C at 3 min and remained stable to ensure that no additional damage was instigated to the skin (Figure 8G-H). On day 7, Co-application of SiO_2_@CIN@CuS starch hydrogel and NIR light group exhibited more angiogenesis and faster wound healing than other groups, further demonstrating the effectiveness and usefulness of low-temperature PTT.

## Conclusion and outlook

This review discussed the mechanism of copper involvement in wound healing, the antibacterial mechanism, and the application in wound healing of copper-containing biomaterials. In the last two decades, progress has been made in the antibacterial mechanism of copper and its modulation of inflammation, angiogenesis, collagen deposition, matrix remodeling, and re-epithelialization, laying an important foundation for the use of copper-doped biomaterials in wounds. Although copper-containing biomaterials have shown good promotion of wound healing in animal models, the following issues still need to be addressed before clinical application:

(1) Dosage and controlled release of copper: While high concentrations of copper enhance the antibacterial effect, there is a risk of inhibiting the proliferation of endothelial cells and fibroblasts. Based on existing studies, controlling local Cu^2+^ in the range of 40-220 μM would allow wounds to benefit from both antibacterial and cell proliferation promotion. Some copper-containing biomaterials use copper as a dopant in bioactive glasses, polymers, and hydrogel scaffolds to accelerate wound healing by releasing copper. Although these materials show great potential in wound healing, it should be noted that a large release of copper from the material in a short period would greatly reduce its biocompatibility. The amount of Cu^2+^ released from these copper-containing biomaterials is closely related to the amount of copper contained in the material, the type of copper contained, and the structure of bioactive glass, polymer, and hydrogel scaffolds. Reducing the amount of copper in the material, adding insoluble Cu or CuO instead of Cu^2+^, and adding copper to bioactive glasses that degrade slowly are simple and effective ways to control the release of copper. In addition, given that chronic wounds have microenvironmental characteristics of low pH, high glucose, high ROS and specific enzyme expression, constructing wound microenvironment-responsive copper-containing wound dressings would contribute to on-demand release of Cu^2+^, improved drug utilization and reduced toxic side effects. It is worth noting that, compared to the wound microenvironment stimuli-responsive drug delivery, photothermal-modulated drug delivery is an active and more controllable approach, which deserves more attention in subsequent studies.

(2) Biosafety: Current studies assessing the biosafety of copper-containing materials are based on organ pathology and blood tests after 2-3 weeks of treatment, and all have concluded that these copper-containing biomaterials have good biocompatibility. However, such assessment methods are inadequate. Before copper-containing biomaterials are introduced into the clinic, their long-term biosafety as well as their absorption, distribution and excretion characteristics in the body should be further explored. In addition, although PTT has been considered very promising in recent years, the cytotoxicity caused by the heat generated by PTT still needs to be considered by researchers. Selecting light with wavelengths located in bio-transparent windows, lowering the excitation power, shortening the irradiation time, and increasing material's photothermal conversion efficiency can help to avoid damage caused by phototherapy. Some studies have explored the conversion of exogenous H_2_O_2_ into highly toxic ·OH through Fenton-like reactions or POD-like activities to enhance antibacterial ability. Although these studies have demonstrated excellent antibacterial effects, there is a lack of in vitro research on the cytotoxicity of normal cells after simultaneous addition of copper-containing materials and exogenous H_2_O_2_. It is worth noting that copper is known for its efficient generation of H_2_O_2_, and this strategy of adding exogenous H_2_O_2_ may result in a large amount of ·OH within a short period of time. Therefore, it may be necessary to control and monitor the ·OH generated in these systems. Recently, the discovery of the mechanism of cuproptosis has also sparked discussions about the biosafety concerns associated with copper again. Whether the toxicity caused by excessive copper application in wounds is related to cuproptosis in wound healing-related cells, and whether copper deprivation strategies can be employed to mitigate copper overload in these cells and rescue them from improper copper application, will require further investigation in the future.

(3) Molecular mechanisms: Although copper has been shown to be involved in several aspects of wound healing, however, some specific processes remain unclear, such as how copper promotes wound contraction and how copper regulates macrophage M2 polarization. Furthermore, although some studies have demonstrated the role of copper in the regulation of certain key cellular signals by conventional protein expression analysis methods, for example, copper was shown to up-regulate HIF-1α expression to promote endothelial cell angiogenesis by western blot. However, this is not sufficient. In subsequent research, cutting-edge techniques such as single-cell transcriptomics, proteomics, and metabolomics should be employed to comprehensively uncover the molecular regulatory mechanisms of copper.

(4) Future directions and clinical translation: The efficacy of copper-containing biomaterials on chronic wounds is currently evaluated in mouse and rat models. However, there are differences between rodents and humans. For example, some diabetic wounds show a tendency to heal without intervention, so it is uncertain whether these biomaterials can achieve the same efficacy in humans. To obtain more accurate results, it may be necessary to seek animal models whose physiological and structural characteristics are closer to those of humans. In addition, some studies may adopt complex processes in the pursuit of multifunctionality, which can lead to difficulties for industrial mass production and high costs, as well as difficulties in ensuring consistency of efficacy from batch to batch. Some degradable nanomaterials have not been designed with sufficient consideration of clinical needs, and these nanomaterials are not suitable for prolonged preservation and transportation after production because of their instability, which greatly limits their clinical translation. Another thing that should not be overlooked is that the wound dressing obtained should be simple to use and avoid repeated manipulations, as this will help to minimize secondary injuries and improve patient compliance.

In conclusion, the advances made in wound therapy with copper-containing biomaterials are exciting. For copper-containing biomaterials aiming for clinical translation, more systematic and scientific approaches should be employed to assess efficacy and biocompatibility, while striving towards simplicity in preparation, ease of large-scale production, product stability, and economic feasibility. Only then will these materials be accepted by the market and ultimately benefit patients.

## Figures and Tables

**Table 1:** Copper-containing nanozymes for wound healing.

Nanozymes	Enzymatic activities	Performances	Applications	Ref
Cu-HCSs and CuO-HCSs	POD, CAT, SOD	Release of Cu^2+^, ROS production for antibacterial	*S. aureus*-infected wound	12
CuCo_2_S_4_	POD	Used in cooperation with H_2_O_2_ to produce ROS for antibacterial and anti-biofilm formation	MRSA-infected burn wound	118
Cu_5.4_O USNPs	CAT, SOD, GPx	ROS scavenging	Diabetic wound	67
Cu_2_O/Pt nanocubes	OXD, POD, CAT	Co-used with H_2_O_2_ to produce ROS for antibacterial; released Cu^2+^ to promote wound healing	*S. aureus*-infected wound and diabetic wound	120
Cu-PBG	POD	ROS from POD-like activity of Cu-PBG for antibacterial and anti-biofilm effects	*S. aureus*-infected wound and *E. coli*-infected wound	119
CS-Cu-GA NCs	OXD, POD	Generation of ROS in synergy with chitosan, Cu NPs, and Cu^2+^ for antibacterial activity	*S. aureus*-infected wound	45
Cu-CDs	POD, CAT	ROS were generated for antibacterial and anti-biofilm	*S. aureus*-infected wound and mixed bacterial-infected wound	121
AuNPs/Cu-MOFNs	POD	Plasmonic nanozymes enhanced POD-like activity via LSPR to produce more ROS for antibacterial	*S. aureus*-infected wound	122

**Table 2:** Copper-based photothermal materials for wound healing.

Photothermal materials	Performances	Laser	Applications	Ref
PATA3-C4@CuS	Targeting bacteria via electrostatic action; PTT, PDT	980 nm, 1.5 W/cm^2^	Infected wounds (bacterial type not reported)	174
GO-Tob@CuS	Targeting bacteria via electrostatic action; PTT, PDT	980 nm, 1.5 W/cm^2^	*S. aureus* and *P. aeruginosa* mixed infected wounds	55
CuS-PNIPAM	Adherence to bacteria by hydrophobic action of PNIPAM; PTT; controlled release of Cu^2+^	808 nm, 2.0 W/cm^2^	*S. aureus*-infected wound	15
CSO@PM	PM-mediated targeting of bacteria and adsorbed toxins for anti-inflammation; PTT	808 nm, 1.5 W/cm^2^	lipopolysaccharides (LPS)-treated wound, *P. aeruginosa*-infected wound	175
G@CuS	Bacterially expressed gelatinases mediate Cu^2+^ release, PTT, and PDT	808 nm, 1.8 W/cm^2^	*S. aureus*-infected wound	176
BACA/CuNPs/GelMA	PTT, photothermal modulation of Cu^2+^ release	808 nm, 1.2 W/cm^2^	*S. aureus*-infected wound	168
CuS/mSiO_2-_MPS/poly(NIAAm-co-AAm)	PTT, PDT, photothermal modulation of Cu^2+^ release	808 nm, 2.0 W/cm^2^	*S. aureus*-infected wound	154
CuS NDs	PTT, PDT, photothermal modulation of Cu^2+^ release	808 nm, 2.5 W/cm^2^	MRSA-infected diabetic wound	54
AuAgCu_2_ONS	PTT, photothermal modulation of Cu^2+^ and Ag^+^ release	808 nm, 1.5 W/cm^2^	MRSA-infected wound	177
Cu_x_O-PDA	Targeting bacteria via electrostatic action, PTT, and POD-like activity	800 nm, 1.5 W/cm^2^	*S. aureus*-infected wound	178
Cu SASs/NPC	PTT, POD-like activity, GSH consumption via GPx-like activity,	808 nm, 1.0 W/cm^2^	MRSA-infected wound	49
CuMnO_2_NFs	Targeting bacteria via electrostatic action; PTT, POD-like activity	808 nm, 2.0 W/cm^2^	*S. aureus*-infected wound	179
CuSNPs-HA-Fe^3+^-EDTA hydrogel	PTT, Fe^2+^ underwent Fenton reaction	808 nm, 0.5 W/cm^2^	*S. aureus*-infected wound	180
SiO_2_@CIN@CuS	Targeting bacteria via electrostatic action; antioxidant, PTT, photothermal regulation of CIN release	980 nm, 1.0 W/cm^2^	*S. aureus*-infected wound	14
Cu_3_SnS_4_ nanoflakes	Targeting bacteria via electrostatic action, antibacterial via release of Cu^2+^, PTT and PDT	808 nm, 1.0 W/cm^2^	MRSA-infected wound	181
CS-PLA/PCL	PTT, the release of Cu^2+^	808 nm, 0.4 W/cm^2^	The wound of tumor-bearing mice, diabetic wound	182
BSA-BiZ/Cu_x_S NC	PTT, PDT	808 nm, 1.2 W/cm^2^	MRSA-infected wound	183

## Abbreviations

ROS: reactive oxygen species
NPs: nanoparticles
GSH: glutathione
POD: peroxidase
OXD: oxidase
CAT: catalase
SOD: superoxide dismutase
GPx: glutathione peroxidase
PTT: photothermal therapy
PDT: photodynamic therapy
NIR: near-infrared
VEGF: vascular endothelial growth factor
FGF: fibroblast growth factor
MMP: matrix metalloproteinases
IL: interleukin
TNF: tumor necrosis factor
TIMP: tissue inhibitor of metalloproteinase
LOX: lysyl oxidase
HIF: hypoxia-inducible factor
ANG: angiogenin
MOF: metal-organic framework
NO: nitrogen monoxide
PCL: polycaprolactone
EPS: extracellular polymeric substances
MBG: mesoporous bioactive glass
CA: cellulose acetate
PVA: polyvinyl alcohol
HA: hyaluronic acid
PEG: polyethylene glycol
LCST: lower critical solution temperature
GOD: glucose oxidase
LSPR: localized surface plasmon resonance
PNIPAM: poly(N-isopropylacrylamide)
PDA: polydopamine
